# Honey, Wound Repair and Regenerative Medicine

**DOI:** 10.3390/jfb9020034

**Published:** 2018-05-08

**Authors:** Simona Martinotti, Elia Ranzato

**Affiliations:** 1 DiSIT-Dipartimento di Scienze e Innovazione Tecnologica, University of Piemonte Orientale, Viale Teresa Michel 11, 15121 Alessandria, Italy; simona.martinotti@uniupo.it; 2 DiSIT-Dipartimento di Scienze e Innovazione Tecnologica, University of Piemonte Orientale, Piazza Sant’Eusebio 5, 13100 Vercelli, Italy

**Keywords:** honey, scaffold, wound repair mechanisms

## Abstract

Honey possesses anti-bacterial, anti-inflammatory and other properties that are useful for wound healing and tissue regeneration. Furthermore, honey has been used for millennia in folk medicine. The misuse of antibiotics has again boosted the use of honey in regenerative medicine. The multifaceted properties of honey could possibly be exploited for scaffold applications in tissue healing.

## 1. Introduction

Honey possesses a long and intriguing history. In particular, this story is intertwined with that of man’s evolution [1]. In fact, since the ancient times, humankind has used and continues to use bee products both as food and as a useful substance for health [2]. Honey has been described in a number of ancient texts as a wound healing agent either on its own or in combination with other ingredients [1]. In the ancient times, people used honey in combination with vegetable or animal fat, but also as part of an ointment that was used to dry out wounds and prevent suppuration [3]. 

Honey is a viscous, aromatic, sweet food, which contains more than 200 substances [4].

It is primarily composed of sugar (about 76%), water (less than 20%) and other ingredients. The sugar content is responsible for the main characteristics of honey (sweetness). Most of the carbohydrates present in honey are monosaccharides, with more fructose present than glucose. Sucrose is the third most common monosaccharide found in honey. The other disaccharides that are present in honey in very small quantities are maltose, isomaltose, nigerose, turanose and maltulose. The sugars and other components of honey can change during storage. Some of these products of sugar degradation, such as 2-acetylfuran, isomaltol, 3,5-dihydroxy-2-methyl-5,6-diidropiran-4-one and maltol, are formed when exposed to heat in the presence of amino acids, in which contributes to the changes in the color, taste and odor of honey.

The moisture content of honey is one of its most important characteristics, influencing physical properties of honey, such as viscosity and crystallization, and other parameters, including the color, flavor, taste, specific gravity, solubility and conservation [5,6].

Honey also consists of other substances, such as proteins (enzymes), organic acids; vitamins (especially vitamin B6, thiamine, niacin, riboflavin and pantothenic acid); minerals (including calcium, copper, iron, magnesium, manganese, phosphorus, potassium, sodium and zinc); pigments; phenolic compounds; organic acids; a large variety of volatile compounds; and solid particles derived from honey harvesting [6].

## 2. Antibiotic Properties of Honey

The antibacterial properties of honey are typically associated with two main mechanisms:

1. Inhibition of the microbial growth by hydrogen peroxide (H_2_O_2_), which is produced by enzymatic activity (e.g., glucose oxidase); and
2. Inhibition of the microbial growth through non-peroxide activities. These non-peroxide activities rely mainly on the action of complex phenols and organic acids, which are referred to as flavonoids. The most direct evidence for the existence of non-peroxide antibacterial factors in honey is seen in the reports of persistent activity in honey products treated with catalase to remove the hydrogen peroxide [7]. These antibacterial activities rely on the floral source collected by honeybees and therefore, they are not present in all honey products [8].

Honey products with non-peroxide antimicrobial activities are associated with a floral source and are generally derived from the *Leptospermum* species, although this type of activity has also been found in a small number of honey types that were not derived from the *Leptospermum* species [9]. The compound that is primarily responsible for the non-peroxide activity of manuka honey is methylglyoxal (MGO), which is derived from dihydroxyacetone, a compound that is present in high levels in manuka nectar [10]. The reasons for the varying dihydroxyacetone levels in different plants are not yet understood.

Moreover, some studies [11,12,13] have attributed the antimicrobial properties of honey to a combination of different characteristics, such as low pH and high osmolarity, while other researchers [14,15] have recognized the beneficial effects of honey in the presence of some volatile substances.

We also know that honey contains lysozyme, which is a powerful antimicrobial agent. The possibility that the heat-stable non-peroxide antibacterial activity relies on lysozyme was excluded by the finding that the honey used in the study had no detectable activity in a standard test for lysozyme. Lysozyme has been identified in honey products, usually occurring at a level of 5–10 mg/mL and occasionally found at a level of 35–100 mg/mL (expressed as the concentration of egg-white lysozyme of equivalent activity) if the honey is freshly extracted from the comb. This level was found to be much lower in older samples [16].

During the early 2000s, it has been proven that honey works by inhibiting the growth of both anaerobic and aerobic bacteria as well as both gram-positive and negative bacteria [17]. By combining these observations, it is clear that honey possess both bacteriostatic and bactericidal activity against many pathogens [13].

Manuka honey, which is produced by the flowers of *Leptospermum scoparium*, a New Zealand plant, deserves special attention for its biological properties, especially its antimicrobial and antioxidant capacities. It combines the hydrogen peroxide activity with non-peroxide activity against bacteria. This provides it with unique antibacterial properties. It has been demonstrated that the pronounced antibacterial activity of manuka honey directly originates from the MGO that it contains [18,19]. This non-peroxide antibacterial activity due to the presence of MGO is called the unique manuka factor (UMF) [18]. A range of bacteria has been found to be sensitive to manuka honey [19]. Interestingly, no resistant bacteria (*Escherichia coli*, *MRSA*, *Pseudomonas aeruginosa* and *Staphylococcus epidermidis)* have been isolated after the exposure of wound isolates to sub-inhibitory concentrations of manuka honey [19].

## 3. Honey and Wound Repair

Since ancient times, honey has been considered to be beneficial for health and in fact, has often been used in medicine. Wound healing was probably its first use in the medical field (as suggested by some Sumerian writings dated to 2000 A.C.).

Honey was used to treat wounds and intestinal disorders by the ancient Egyptians, Assyrians, Chinese, Greeks and even the Romans [20].

An ancient Egyptian text, the “Smith papyrus” (dated between 2600 and 2200 A.C.), prescribes a mixture of honey and vegetable fibres as a treatment for wounds. In ancient Rome, the historian Plinio suggested that honey could be used to treat abscesses in the mouth, while a mixture of honey and fish fat could treat wounds. 

Trotula de Ruggiero of the Salernitan Medical School recommended honey as a moisturizer, hair dye and facial mask ingredient [21]. 

The use of honey as a medicine and ointment has continued to this day [3]. However, the leading role of honey in wound care has rapidly declined after the discovery of antibiotics. The misuse of antibiotics, with the consequent onset of resistance, has allowed the honey to regain the role of an antibacterial agent, especially when considering that honey does not have any toxicity in human tissues [22].

Recently, the use of honey as a medicine has been rediscovered both in in vitro studies and in clinical trials [23]. In recent years, honey has been reconsidered as an antibacterial agent for the treatment of ulcers, wounds and other infections [24].

## 4. Honey and Scaffolds Used for Regenerative Medicine

A wound is the destruction (accidental, violent or surgical) of the integrity of a tissue, with damage also inflicted on the neighbouring areas [3]. Honey has been known since ancient times as an agent that is able to encourage the repair of wounds, guaranteeing healing with little or no scar formation [3]. The viscosity of honey is particularly important (which varies according to the type of honey used) as it allows the honey to constitute a protective barrier that prevents infections in the wounds. The high content of sugars can improve the local nutrition of the damaged areas [1]. Honey also facilitates the deodorization of infected wounds, as it provides an alternative to the metabolism of amino acids and dead cells to bacteria [25]. According to some authors, the low pH of honey could help to create and maintain optimal conditions for fibroblasts, which require an acidic environment for activities, such as migration and the organization of collagen [26]. Honey also produces H_2_O_2_, which can be dangerous for cells and tissues in excessive quantities, but at physiological concentrations, this hydrogen peroxide acts as a “messenger” modulating the different cell signalling pathways involved in the repair of wounds [27]. There are various data indicating that the rates of H_2_O_2_ production in honey can vary greatly [28].

H_2_O_2_ produced by honey exerted bacteriostatic and DNA-degrading activities on the bacterial cells. The extent of the damaging effects of the H_2_O_2_ from honey is affected by the bacterial sensitivity to oxidative stress, the growth phase and their survival strategy (non-spore forming versus spore forming species) as well as by the modulation of other honey compounds [29].

One of the crucial phases of the wound repair process is inflammation [30]. Honey can both promote and repress the inflammatory process [31]. With the plethora of its components, honey is able to either stimulate or inhibit the release of certain cytokines (tumour necrosis factor-α, interleukin-1β and interleukin-6) from human monocytes and macrophages, which depends on the wound conditions. Similarly, honey can reduce or activate the production of reactive oxygen species from neutrophils, which also depends on the wound microenvironment. The immunomodulatory activity of honey is highly complex because of the involvement of multiple quantitatively variable compounds among honey of different origins [31].

In honey, we can find various compounds, of plant origin or derived from the metabolism of the same bees, with immunomodulatory effects, which can influence the wound healing process. Some studies have investigated the effect of honey directly on the biology of skin cells. The treatments with honey have little toxic effect on the keratinocytes and fibroblasts, which are the main cells of the skin [32]. Therefore, it was demonstrated that honey is not toxic and can be used for both external application on intact ski and as dressings on wounds. In addition, the data regarding wounds and cell migration assays showed that honey induces a marked increase in the regenerative capacity of skin cells [33]. Therefore, honey is able to promote re-epithelialization [22], which involves the cell migration triggered by the stimulus induced by a lesion. This process is known as the epithelial–mesenchymal transition (EMT). Upon exposure to honey, the keratinocytes underwent changes in the expression of the regulatory genes of EMT, with variations related to the type of honey used [32]. The extracellular matrix is particularly important in the wound closure processes and after exposure to honey, the keratinocytes increase the production of matrix metalloproteases (MMPs), such as MMP-9, and influence the degradation of type IV collagen [34]. Martinotti et al. [35] also found the biological activity of honey from honeydew on keratinocytes. 

Tissue engineering represents the first interdisciplinary sector that integrates almost all fields of science, such as cell/molecular biology, materials science, chemistry, physics, industrial engineering and medicine. This discipline represents the new frontier in the biomedical field, with the aim of repairing or even replacing damaged tissues and organs (such as muscles, bones, cartilages, etc.) from diseases, trauma or aging by restoring their integrity and functionality. This approach, which is also called regenerative medicine, is revolutionizing medicine, paving the way for new possibilities of care and a better quality of life for patients. The so-called “weapons” of regenerative medicine are stem cells, growth factors and scaffolds. Stem cells are the progenitor cells of all the organs and tissues of living beings. Under certain conditions, the stem cells can differentiate and develop into any cell type and tissue. The appropriate growth factors, which are specific for each type of cell and tissue, can be selected to promote and accelerate the process of cell differentiation and proliferation. Finally, the scaffolds represent the 2D or 3D support, which must be able to host and promote the growth and differentiation of stem cells. The scaffolds must be projected and realized with different materials, several architectures and geometries in order to promote the regeneration of a tissue or organ. Apart from the chemical composition, the scaffold structure plays a fundamental role in the development of the tissue. The constituent material represents a temporary structure where the cells can promote regeneration and subsequently, tissue restoration. A scaffold needs to fulfil specific criteria, such as suitable mechanical properties, degradation times and bioactivity. 

Scaffolds should provide support for either extraneously applied or endogenous cells to attach, grow and differentiate during both the in vitro culture and in vivo implantation. The biomaterials used to fabricate the scaffolds need to be compatible with the cellular components of the engineered tissues and endogenous cells in host tissue. Scaffolds are not only supports but they may interact with the cell components of the tissues to actively facilitate and regulate their activities. The scaffold may also be useful as a delivery vehicle or reservoir for exogenous growth-stimulating signals, such as growth factors, to speed up regeneration. 

To achieve this aim, the biomaterials and scaffold need to be compatible with the molecules and easy to manipulate for encapsulation for controlled release.

Some natural products are of relevant interest for increasing the bioactivity of scaffolds in order to increase the cell infiltration and proliferation [36].

The multifaceted characteristics of honey in terms of the anti-inflammatory activity, wound repair efficacy and antibacterial properties has attracted the attention of regenerative medicine researchers (see Table 1). 

Minden-Birkenmaier et al. characterized the manuka honey-containing poly(e-caprolactone) (PCL) nanofiber scaffolds with regards to wound healing. They showed that honey positively increased the entry of fibroblasts into the scaffold, while also inhibiting the growth of Gram-negative *Escherichia* *coli* [37].

Another conference paper presented [38] data concerning the spinnability of honey–alginate formulations, which also characterized the engineered scaffolds in terms of cell viability, adherence and molecular expression of keratinocyte and fibroblast cell lines. The higher attachment rate of the skin cells on the honey-prepared material is also correlated to a higher expression of collagen I and III and more E-cadherin expression, suggesting the superiority of the honey-based scaffold.

Maleki et al. [39] examined the nanofiber meshes produced from poly(vinyl alcohol) (PVA) and honey via electrospinning. Dexamethasone sodium phosphate, a model anti-inflammatory drug, was incorporated into the fibres to provide anti-inflammatory properties in the early stages of treatment. This study demonstrated that an anti-inflammatory drug can efficiently be released and honey can be used as a natural antibiotic to increase the wound dressing efficiency, thus enhancing the repair rate. 

Hixon et al. [40] compared silk fibroin cryogels and electrospun scaffolds incorporated with a 5% manuka honey. They used different manuka honey samples from New Zealand, with different Unique Manuka Factors (UMF) [41]. The UMF reflects the antimicrobial efficacy of a honey. The results showed that the engineered scaffolds possess the same properties and effects on bacteria, regardless of a high or low UMF value. 

The same group [42] explored the use of cryogels, which are scaffolds possessing high porosity, mechanical stability and a sponge-like consistency. This study used varying amounts of manuka in the cryogel scaffolds. The authors determined that a 5% manuka silk fibroin cryogel has the potential to inhibit bacterial growth, while still maintaining adequate porosity, mechanical properties, and cell infiltration. 

## 5. Conclusions

A variety of engineered scaffolds has been realized for tissue engineering using different materials. The use of a single growth factor or a mixture of growth factors can improve the biocompatibility of a scaffold for superior tissue regeneration. The preliminary data about the scaffolds incorporating honey products provide the rational for the use of this natural product in an increasing number of applications, including tissue repair and regeneration. However, other studies on the active components of honeys are needed in order to determine single effective molecules and synergistic interactions between the vast number of active compounds that mediate their biological effects.

## Figures and Tables

**Table 1:** Studies carried out on honey-based scaffolds.

Scaffold Type	Honey Type	Type of Application
Poly(e-caprolactone) (PCL) nanofiber [37]	Manuka	Cellular proliferation (fibroblasts) and anti-microbial efficiency (*Streptococcus agalactiae* and *Escherichia coli*)
Honey-alginate formulation [38]	Not defined	Cellular proliferation (3T3 fibroblasts and HaCaT keratinocytes)
Aqueous mixtures of poly(vinyl alcohol) (PVA) [39]	Iran-Tabriz honey	Anti-inflammatory drug release
Silk fibroin cryogels [40]	Manuka	Anti-microbial efficiency (*Escherichia coli*, *Streptococcus agalactiae*, *Staphylococcus aureus*)
Silk fibroin cryogel [42]	Manuka	Cellular proliferation (osteosarcoma-derived MG-63 cells) and anti-microbial efficiency (*Escherichia coli*, *Streptococcus agalactiae*, *Staphylococcus aureus*)
